# Clinical application of noninvasive prenatal testing in the detection of fetal chromosomal diseases

**DOI:** 10.1186/s13039-021-00550-5

**Published:** 2021-06-14

**Authors:** Yu Pang, Chaohong Wang, Junxiang Tang, Jiansheng Zhu

**Affiliations:** 1 Affiliated Maternity and Child Health Hospital of Anhui Medical University, Hefei, China; 2 Maternity and Child Health Hospital of Anhui Province, Hefei, China

**Keywords:** Noninvasive prenatal testing, Chromosome aneuploidy, Copy number variation, Chromosomal microarray analysis

## Abstract

**Objective:**

To assess the detection efficiency of noninvasive prenatal testing (NIPT) for fetal autosomal aneuploidy, sex chromosome aneuploidy (SCA), other chromosome aneuploidy, copy number variation (CNV), and to provide further data for clinical application of NIPT.

**Materials and methods:**

25,517 pregnant women who underwent NIPT testing in Anhui Province Maternity and Child Health Hospital from September 2019 to September 2020 were selected, and samples with high-risk test results were subjected to karyotype analysis for comparison by using amniotic fluid, with some samples subjected to further validation by chromosomal microarray analysis, and followed up for pregnancy outcome.

**Results:**

A total of 25,517 pregnant women who received NIPT, 25,502 cases were tested successfully, and 294 high-risk samples (1.15%) were detected, there were 96 true positive samples, 117 false positive samples and 81 cases were refused further diagnosis. Samples with high risk of autosomal aneuploidy were detected in 71 cases (0.28%), and 51 cases were confirmed, including: trisomy 21 (T21) in 44 cases, trisomy 18 (T18) in 5 cases, and trisomy 13 (T13) in 2 cases; the positive predictive value (PPV) was 91.67%, 45.45%, and 33.33%, respectively, and the negative predictive value was 100%, the false positive rate (FPR) was 0.02%, 0.02%, and 0.02%, respectively.13 samples with high risk of mosaic trisomies 21, 18, and 13 were detected, and 1 case of T21mos was confirmed with a PPV of 8.33%. Samples with high risk of SCA were detected in 72 cases (0.28%), and the diagnosis was confirmed in 23 cases, with a PPV of 41.07% and a FPR of 0.13%. These included 3 cases of 45,X, 6 cases of 47,XXY, 8 cases of 47,XXX and 6 cases of 47,XYY, with PPVs of 12.00%, 50.00%, 72.73%, and 75.00%, respectively, and false-positive rates of 0.09%, 0.02%, 0.01% and 0.01% respectively. Samples with high risk of CNV were detected in 104 cases (0.41%) and confirmed in 18 cases, with a PPV of 32.14% and a FPR of 0.15%. Samples with high risk of other chromosomal aneuploidy were detected in 34 cases (0.13%), and the diagnosis was confirmed in 3 cases, which were T2, T9, and T16 respectively. The overall PPV for other chromosome aneuploidy was 12.50%, with a FPR of 0.08%.

**Conclusion:**

NIPT is indicated for trisomies 21, 18 and 13 screening, especially for T21. It also has some certain reference value for SCA and CNV, but is not recommended for screening of other chromosomal aneuploidy.

## Background

With the liberalization of birth policy, the number of births in China has gradually increased each year, and the rate of birth defects has also increased. The annual birth defects rate in China is about 5.6%, with a growth of 900,000 cases each year. Among them, Down’s Syndrome (DS) cases increase about adds 23,000–25,000 every year. Chromosomal disease is one of the most common causes of birth defects. At present, prenatal screening is an effective method to prevent birth defects [1]. Traditional serological screening uses pregnant women's serum markers such as AFP, β-hCG, uE3, and inhibin-A to find high-risk pregnant women. However, the accuracy of the screening is not reliable and the rate of missed diagnosis is high [2]. Although amniocentesis and chorionic sampling are more accurate, they can not easily be accepted and promoted, because their invasive operations, can easily cause anxiety, and fear in the pregnant women, and may possibly result in risks of miscarriage and intrauterine infection [3].

In 1997, Professor Yuming Lo of the Chinese University of Hong Kong proved for the first time that there is fetal free DNA in the peripheral blood of pregnant women, derived from the placenta, which open up a new method of noninvasive prenatal testing [4]. So far, it has been widely used in many countries around the world [5]. The noninvasive prenatal testing uses a new generation of high-throughput sequencing technology to detect free fetal DNA based on maternal plasma. After bioinformatics analysis, the fetal chromosomal abnormalities are detected, which proves that NIPT tend to be noninvasive, safe, and fast, with high accuracy and reliability. NIPT not only has high sensitivity and specificity for autosomal aneuploidy diseases but also has certain clinical practical value for sex chromosome aneuploidy, other chromosome aneuploidy, and chromosome submicroscopic structure (copy number variation, CNV). Screening has certain clinical practical value [6]. This study selected 25,517 pregnant women who underwent NIPT screening to analyze the effectiveness of NIPT in screening fetal chromosomal abnormalities and explore its clinical application value.

## Materials and methods

### Subjects

25,517 pregnant women who underwent NIPT testing in Anhui Province Maternity and Child Health Hospital from September 2019 to September 2020, aged 16–48 years, with 11^+5^ to 30^+6^ weeks' gestation and had singleton pregnancies, were selected. The indications include: serologic prenatal screening for high and critical risk, i.e., high risk for T21 risk value ≥ 1/270, high risk for T18 risk value ≥ 1/350, critical risk for T21 risk value = 1/270–1/1000 and T18 risk value = 1/350–1/1000; high risk, old pregnant women who missed Down’s Syndrome screening, pregnant women who voluntarily underwent NIPT without indication; ultrasound fetal abnormalities suggested by ultrasound include nuchal translucency (NT) greater than 3 mm, ventricular bright spot, abnormal fetal choroid plexus echogenicity, slightly widened lateral ventricles, and enhanced renal and intestinal echogenicity. Pregnant women with twin or multiple pregnancies, chromosomal abnormalities, in vitro fertilization (IVF), history of blood transfusion within 2 years, history of stem cell treatment and transplantation were excluded as factors that might have an impact on NIPT results. All pregnant women were informed about the need for prenatal diagnosis, the indications for NIPT screening and its limitations and associated risks. All pregnant women who participated in this study signed an informed consent form.

### NIPT screening

Maternal peripheral blood samples (10 mL) were collected in a Cell-Free DNA BCTTM tube (Streck company, USA), whole blood was centrifuged at 1600 × g for 10 min at 4 °C, and then transferred into a new 2.0 ml centrifuge tube, where it was centrifuged at 1600×*g* for 10 min, the maternal plasma was stored in − 80 °C refrigerator until DNA extraction, fetal DNA was extracted by the QIAamp Circulating Nucleic Acid Kit (Qiagen), library construction, the Illumination NextSeqCN500 sequencer was used to perform high-throughput sequencing, bioinformatics analysis, compared with the reference sequence map of human genome, and used noninvasive prenatal screening analysis software Illumina Sequencing Analysis Viewer 1.9.1 to calculate the risk rate and Z score of chromosomes, − 3 < Z score < 1.96 was considered low risk, Z score = 1.96 ~ 3 was considered gray zone, Z score ≥ 3 or Z score ≤ − 3 was considered high risk [7], the calculation of fetal fraction (FF) was divided into two parts, male fetal fraction was estimated according to the content of Y chromosome, while female fetal fraction was estimated based on fragment size distribution of cell-free DNA, the detection threshold of fetal fraction was set to 4%, and Z score can be calculated only when fetal fraction was greater than or equal to 4%. If it was lower than the threshold, blood collection was needed again [8], all pregnant women with a high risk of NIPT results were received genetic counseling, and interventional prenatal diagnosis was selected to verify the NIPT results.

### Karyotype analysis

Amniotic fluid samples were collected from pregnant women who were suggestive of high risk after NIPT testing, and an interventional prenatal diagnosis protocol was selected: amniocentesis was performed under ultrasound guidance, 20 mL to 30 mL of amniotic fluid was extracted and cultured at 37 °C in a CO_2_ constant temperature incubator to harvest cells in the peak of division, standardized cell culture, filmmaking, G-banding, and fetal karyotype analysis, and the karyotype was analyzed according to the standard of ISCN (2016) [9].

### Chromosome microarray analysis

For pregnant women with NIPT testing results of CNV, amniotic fluid chromosome microarray analysis was selected: amniotic fluid specimens were centrifuged, precipitated, and the genomic DNA were extracted, and SNP array assay was performed according to operation procedures of chip of Affymetrix CytoScan 750 K (Affymetrix, USA) [10], data were collected and results were analyzed, public databases (CLINGEN, DECIPHER, CLINVAR, OMIM, DGV, ISCA, NCBI, UCSC) were used to explain the data. None of the samples were tested using aCGH (array-based comparative genomic hybridization).

### Data analysis and follow-up of pregnancy outcomes

The NIPT testing data were analyzed and the number of true positive cases, false positive cases, true negative cases, false negative cases, PPV, NPV, FPR, sensitivity, and specificity were calculated. PPV = number of true positive cases/(number of true positive cases + number of false positive cases) × 100%, NPV = number of true negative cases/(number of true negative cases + number of false negative cases) × 100%, sensitivity = number of true positive cases/(number of true positive cases + number of false negative cases) × 100%, specificity = number of true negative cases/(number of true negative cases + number of false positive cases) × 100%, false positive rate = number of false positive cases/(number of true negative cases + number of false positive cases) × 100%. Meanwhile, pregnancy outcomes were also followed up by telephone or by reviewing the maternal follow-up registry to record the presence of miscarriage, induction of labor, delivery, and false-negative samples.

## Results

### Screening results

A total of 25,517 samples were collected, and the information collected included sample number, storage temperature, date of blood sampling, age, weight, gestational week at delivery, date of last menstrual period, expected date of delivery, number of pregnancies, presence of Down’s Syndrome screening, whether they had undergone ultrasound, singleton or twin pregnancy, whether they were IVF, pregnancy outcome, previous history of allogeneic blood product transfusion, history of stem cell treatment and transplantation, history of chromosomal disorders in the husband, and registered return telephone numbers and addresses, and telephone callbacks were made to all samples. For the high-risk samples, all of them were called back several times and further prenatal intervention was recommended. No false-negative cases were found after follow-up of samples with low-risk test results of the NIPT, and no false-negative cases were registered by checking the pregnancy follow-up registry.

Among the 25,517 samples, 52 cases needed to be re-collected due to hemolysis, two Z score gray area results, low fetal DNA concentration (FF < 4%), etc. 15 cases were refunded for failure to meet the testing requirements after re-collection. We successfully tested 25,502 samples and screened 294 high-risk chromosomal samples, among which 213 high-risk NIPT samples were selected for interventional prenatal diagnosis, 96 true positive cases were detected, 117 false positive cases were detected, and 81 cases were rejected for further verification.

294 high-risk samples were detected using NIPT (1.15%), including 48 cases of T21, 15 cases of T18, and 8 cases of T13; 72 cases of sex chromosome abnormalities: 33 cases of 45,X, 14 cases of 47,XXY, 17 cases of 47,XXX, and 8 cases of 47,XYY; 104 cases of CNV; and 34 cases of other chromosomal aneuploidies; there were 13 cases of T21mos, T18mos and T13mos.

### Detection efficiency of T21, T18, and T13 in NIPT

The samples with a high risk of T21, T18 and T13 detected by NIPT were subjected to karyotype analysis, and some samples were subjected to karyotype analysis and chromosome microarray analysis, and all samples with T21, T18 and T13 were correctly detected. T21: 48 cases (0.19%), 44 true positive cases; T18: 15 cases (0.06%), 5 true positive cases; T13: 8 cases (0.04%), 2 true positive cases. The PPVs of T21, T18 and T13 were 91.67%, 45.45% and 33.33%, respectively. The specificities of NIPT for T21, T18 and T13 were 99.98%, 99.98% and 99.98%, respectively. The FPR of T21, T18 and T13 were all 0.02%. The negative predictive values of T21, T18 and T13 were all 100%, and no false-negative cases of T21, T18 and T13 were recorded (Table 1). Among the samples with no further karyotype verification for T18 and T13, 4 cases (3 for T8 and 1 for T13) were induced at the local hospital or had spontaneous abortions due to fetal abnormalities or malformations on ultrasound, and 2 cases (1 each for T18 and T13) were induced directly back to the local hospital without genetic and pathological examination of the induced or spontaneously aborted tissues, which is a limitation of this study. All confirmed cases with T21, T18, and T13 screened with NIPT chose to terminate the pregnancy.

**Table 1:** The efficiency of NIPT in screening T21, T18, T13

NIPT results	Positive cases (N)	Amniotic fluid puncture Cases (N)	TP (N)	FP (N)	FN (N)	TN (N)	Sensitivity (%)	Specificity (%)	PPV (%)	NPV (%)	FPR (%)
T21	48	48	44	4	0	25454	100	99.98	91.67	100	0.02
T18	15	11	5	6	0	25491	100	99.98	45.45	100	0.02
T13	8	6	2	4	0	25496	100	99.98	33.33	100	0.02

NIPT, noninvasive prenatal testing; TP, true positive; FP, false positive; FN, false negative; TN, ture negative; PPV, positive predictive value; NPV, negative predictive value; FPR, false positive rate

### Detection efficiency of SCA and CNV in NIPT

For samples with NIPT suggesting high risk of SCA and CNV, karyotype analysis was performed, and some samples were subjected to karyotype analysis and chromosome microarray analysis. 72 cases (0.28%) of SCA and 104 cases (0.41%) of CNV were detected, and no false-negative cases were recorded. The test results were as follows: Among 72 cases with SCA, 23 cases were true positive, and 33 cases were false positive, with a PPV of 41.07% and a FPR of 0.13%. Among them, 33 cases of 45,X including 3 true positive cases and 22 false positive cases, with a PPV of 12.00%; 14 cases of 47,XXY, including 6 true positive cases and 6 false positive cases, with a PPV of 50.00%; 17 cases of 47,XXX, including 8 true positive cases and 3 false positive cases, with a PPV of 72.73%; 8 cases of 47,XYY, including 6 true positive cases and 2 false positive cases, with a PPV of 75.00%. The order of detection efficiency was 47,XYY > 47,XXX > 47,XXY > 45,X. Among the pregnancy outcomes of the 23 true positive SCA samples, 17 were induced, 5 were born, and all the children were phenotypically normal at present, and 1 continued pregnancy was in progress. Among 104 cases with CNV, 18 cases were true positive, and 38 cases were false positive, with a PPV of 32.14% and a FPR of 0.15% (Table 2).

**Table 2:** The efficiency of NIPT in screening SCA and CNV

NIPT results	Positive cases (N)	Amniotic fluid puncture Cases (N)	TP (N)	FP (N)	FN (N)	TN (N)	Specificity (%)	PPV (%)	FPR (%)
SCA	72	56	23	33	0	25446	99.87	41.07	0.13
45,X	33	25	3	22	0	25477	99.91	12.00	0.09
47,XXY	14	12	6	6	0	25490	99.98	50.00	0.02
47,XXX	17	11	8	3	0	25491	99.99	72.73	0.01
47,XYY	8	8	6	2	0	25494	99.99	75.00	0.01
CNV	104	56	18	38	0	25446	99.85	32.14	0.15

NIPT, noninvasive prenatal testing; SCA, sex chromosome aneuploidy; CNV, copy number variation; TP, true positive; FP, false positive; FN, false negative; TN, ture negative; PPV, positive predictive value; FPR, false positive rate

### Detection efficiency of other chromosomes in NIPT

Whole-genome sequencing was performed using NIPT, and we also analyzed the results of other chromosomal aneuploidy detection by NIPT. 34 cases (0.13%) of other chromosomal aneuploidy abnormalities were detected, the overall PPV for other chromosome aneuploidy was 12.50%. Among them, there were 3 true positive cases: 1 case each of T2, T9 and T16, and their PPVs were 50.00%, 25.00% and 25.00%, respectively. The FPR was 0.08% in 21 false-positive cases, and 10 cases were not validated. Among the false-positive samples, T7 was detected most frequently, followed by T8, T10, and T16, and the rest were detected in very small numbers. Pregnancy outcome: The child of the sample T2 was born with a normal phenotype. The sample T9 chose to induce labor. The sample T16 chose to continue the pregnancy. In addition, 13 cases of mosaic trisomies 21, 18 and 13 with high risk were detected, with a total PPV of 8.33%. One true positive case (sample number 20AH00515) was T21mos. The fetus was born with a normal phenotype. One case was not verified (sample number 19AH03199, direct induction of labor), and the rest were false positives (Table 3).

**Table 3:** The efficiency of NIPT in screening other chromosomal aneuploidies

NIPT results	Positive (N)	TP (N)	FP (N)	Unverified (N)	PPV (%)
T1	1	0	1	0	0
T2	2	1	1	0	50.00
T3	1	0	1	0	0
T7	9	0	3	6	0
T8	5	0	3	2	0
T9	2	1	1	0	50.00
T10	3	0	3	0	0
T12	1	0	1	0	0
T14	1	0	1	0	0
T15	2	0	2	0	0
T16	4	1	3	0	25.00
T17	1	0	0	1	0
T20	2	0	1	1	0
T21mos	5	1	3	1	25.00
T18mos	5	0	5	0	0
T13mos	3	0	3	0	0

NIPT, noninvasive prenatal testing; TP, true positive; FP, false positive; PPV, positive predictive value

### Detection efficiency of NIPT in screening the CNV

**Fig. 1:**
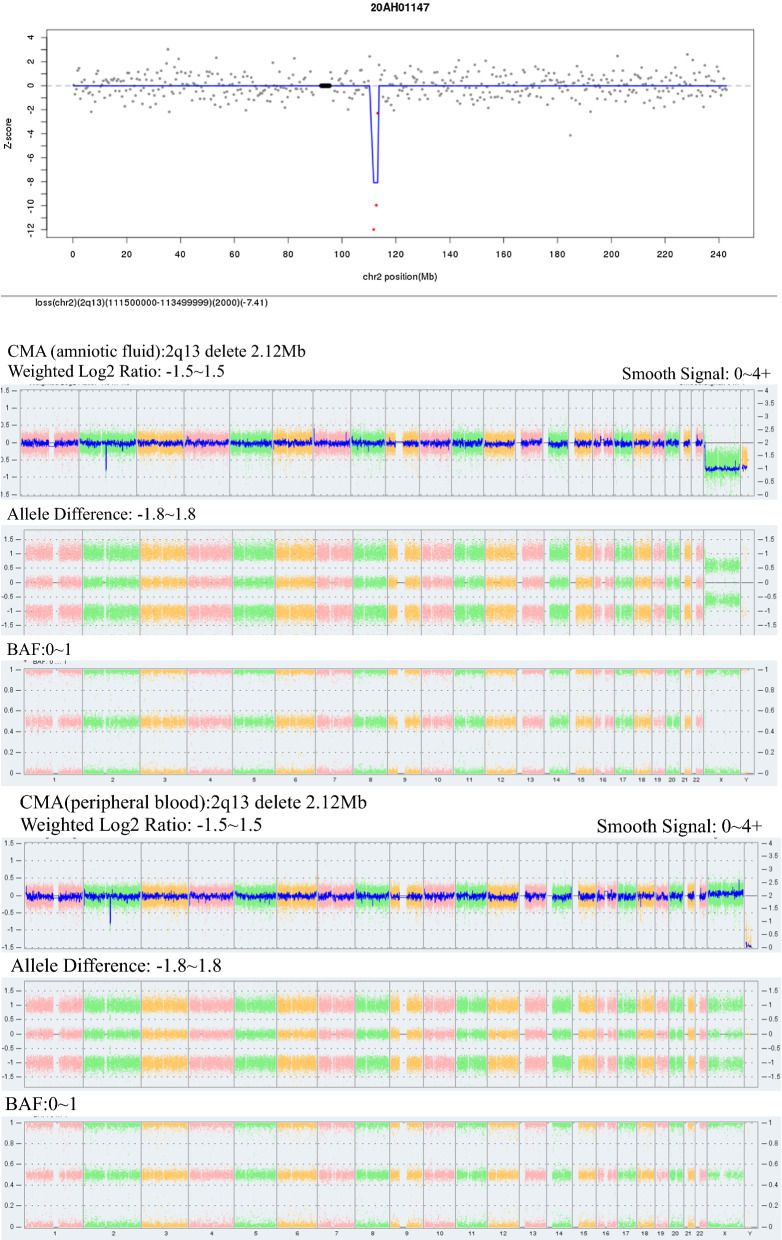
Sample number: 20AH01147. NIPT result:2q13 delete 2Mb

**Fig. 2:**
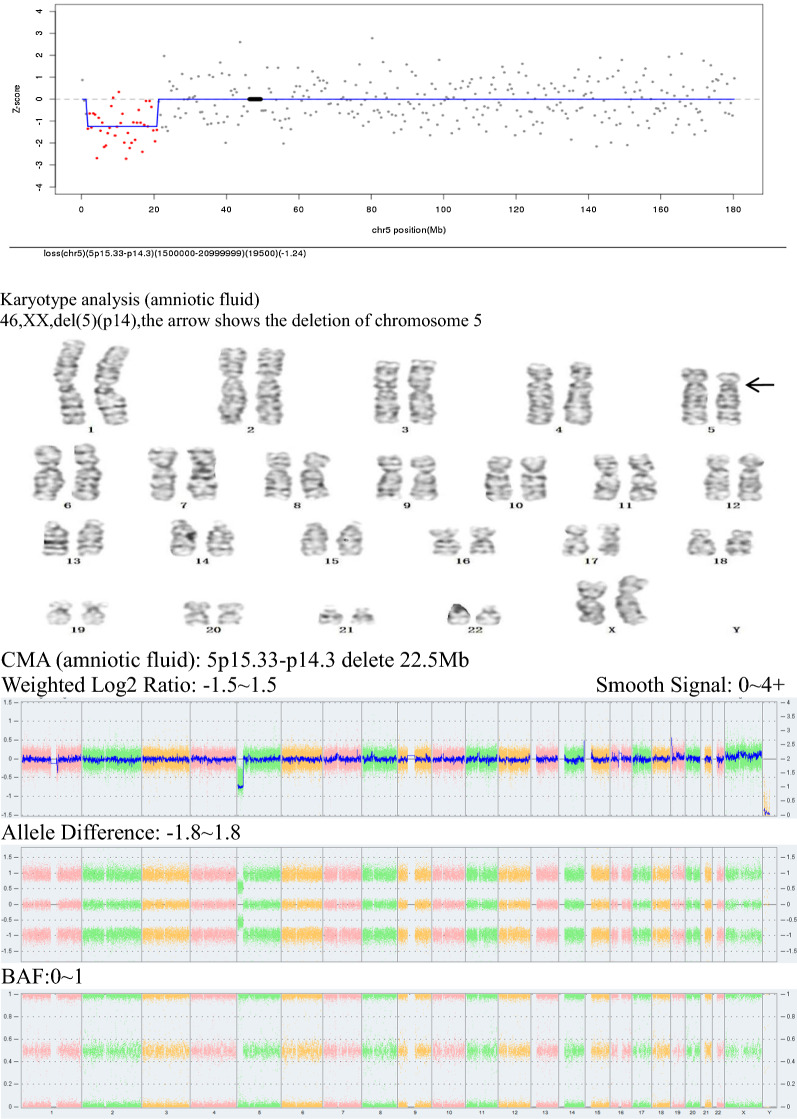
Sample number: 20AH02846 (diagnosed as Cri-du-chat syndrome). NIPT result:5p15.33-p14.3 delete 19.5Mb

Among the 56 CNV samples diagnosed by intervention, 18 were true positive, 11 were microduplicates, 7 were microdeletions, 5 were pathogenic, 4 were probable pathogenic, 9 were of variant of uncertain significance (VOUS), and 1 was a karyotype abnormality. Pregnancy outcome: 4 cases of induced labor; 13 cases of full-term delivery with normal child phenotype; 1 case of continued pregnancy (Table 4, Figs. 1, 2).

**Table 4:** Classification of true positive CNV and pregnancy outcome

No	Sample number	Age (years)	Gestational weeks	NIPT results	Karyotype results	CMA results and classification	Pregnancy outcome
1	19AH03422	30	16^+3^	2q12.1q12.3 duplicate 2.50 Mb	46,XN	2q12.1q12 .3duplicate 2.51 Mb, VOUS	Delivery, normal
2	19AH05194	25	16^+7^	3p26.3 duplicate 2.50 Mb	46,XN	3p26.3 duplicate 2.21 Mb, Likely pathogenic	Delivery, normal
3	20AH01147	35	18^+2^	2q13 lose 2.00 Mb	46,XN	2q13 lose 2.12 Mb, Likely pathogenic	Delivery, normal
4	20AH00928	36	16^+6^	8q23.3-8q24 duplicate 29.00 Mb	Refused	8p23.3 lose 1.86 Mb, 8p23.3p23.1 duplicate 4.92 Mb, 8p23.1p12 lose 24.84 Mb, 8p12q24.3 duplicate 113.39 Mb, Pathogenic	Induced labor
5	20AH02333	26	13^+4^	18q12.3 duplicate 2.00 Mb	46,XN	18q12.3duplicate 1.03 Mb, VOUS	Delivery, normal
6	20AH01774	26	21^+1^	5p15.33-p14.3 duplicate 1.32 Mb, 5q31.1-q35.3 duplicate 1.23 Mb	46,XN	3p26.3duplicate 1.15 Mb, VOUS	Delivery, normal
7	20AH02846	25	17^+6^	5p15.33-p14.3 lose 19.50 Mb	46,XX, del(5) (p14)	5p15.33-p14.3 lose 22.5 Mb, Pathogenic	Induced labor
8	AB198R82670	37	17^+5^	6p12.3-6p12.1 lose 4.60 Mb	46,XN	6p12.36p12.1duplicate 4.36 Mb, VOUS	Delivery, normal
9	AB198R94013	26	18^+1^	18p11.32-18p11.21 lose 14.85 Mb	46,XN	11q21q22.1 duplicate 1.89 Mb, VOUS	Delivery, normal
10	AB19HG00152	32	19^+3^	22q11.21-22q11.21 duplicate 1.35 Mb	46,XN	22q11-21 duplicate 2.35 Mb, Pathogenic	Induced labor
11	AB198R04444	25	18^+1^	17p12 lose 2.50 Mb	46,XN	17p12 lose 1.42 Mb, Pathogenic	Delivery, normal
12	AB198R02692	34	21^+2^	3q26.33-3q27.1 duplicate 3.35 Mb	46,XN	3q26.q27.1duplicate 3.29 Mb, VOUS	Delivery, normal
13	AB198R02616	36	17^+0^	16p13.11-p12.3 duplicate 3.60 Mb	46,XN	16p13.11p12.3 duplicate 2.67 Mb, Likely pathogenic	Delivery, normal
14	AB19HG03892	28	14^+6^	12p11.21-12q12 duplicate 9.85 Mb	46,XN	12p11.21p11.1-12q11q12 duplicate 2.49 Mb, VOUS	Continued pregnancy
15	AB198R02189	38	20^+5^	1q21.1q21.2 duplicate 3.00 Mb	46,XN	1q21.1q21.2duplicate 1.71 Mb, Pathogenic	Induced labor
16	AB198R02191	31	17^+5^	17p13.1-17p12 duplicate 4.60 Mb	46,XN	14q13.1 duplicate 550 kb, VOUS	Delivery, normal
17	20B0012795	20	18^+4^	8p22-p21.3 lose 5.21 Mb	46,XN	8p22p21.3 lose 3.81 Mb, VOUS	Delivery, normal
18	20B0018624	30	17^+2^	13q33.1-q33.3 lose 5.68 Mb	46,XN	13q33.1q33.3 lose 4.66 Mb, Likely pathogenic	Delivery, normal

NIPT, noninvasive prenatal testing; CMA, chromosome microarray analysis; VOUS, variant of uncertain significance

## Discussion

Chromosomal abnormalities are one of the most serious birth defects in newborns. There is no effective treatment available, and prenatal screening is a more effective way to reduce birth defects in affected children. NIPT is an important tool for prenatal screening and was recommended by the International Society for Prenatal Diagnosis (ISPD) and the American College of Obstetricians and Gynecologists (ACOG) in 2012 as a test for people at high risk of chromosomal aneuploidy [11, 12]. In this paper, we analyzed the screening efficacy of NIPT for fetal autosomes, SCA and other chromosomal aneuploidies and CNV in 25,502 pregnant women and investigated its clinical application value.

The range of PPV using NIPT was reported to be 65–94% for T21, 47–85% for T18, and 12–62% for T13 [13–15]. In this study, NIPT suggested 48 cases with high risk of T21 (0.19%), 15 cases with high risk of T18 (0.06%), and 8 cases with high risk of T13 (0.04%). The PPVs of T21, T18, and T13 were 91.67%, 45.45%, and 33.33%, respectively. The negative predictive values of T21, T18 and T13 were all 100%. The range is generally similar to that reported in the above literature. The sequential decrease in PPV was not only dependent on the sensitivity of NIPT but also correlated with the sequential decrease in the incidence of T21, T18, and T13 [16]. Also, since the karyotype of induced or spontaneous abortion tissues was not examined in this study, some true-positive samples for NIPT may have been included, which also affected the PPV. This study showed that the screening efficacy of NIPT for T21, T18, and T13 had high accuracy and reliability, especially for T21 [17].

In addition, 13 cases of T21mos, T18mos and T13mos were detected in this study, with 1 true positive case (T21mos), the overall PPV for T21mos/T18mos/T13mos was 8.33%. Its sample number is 20AH00515, 30 years old, and with 20^+4^ weeks' gestation. Its Down’s Syndrome screening result is: T21, 1/620. NIPT suggested T21mos and high risk (Z-score = 3.63). CMA test showed: arr[GRCH37]21q21.1(19652230–21419497) × 3, suggesting that chromosome 21 of the fetus was 21q21.1, with duplication of 1.76 Mb, involving 1 OMIM gene: TMPRSS15 (606635). After checking databases, no repeated disease was reported, and the clinical significance of this repeat was unclear. After genetic counseling, the case chose to continue the pregnancy and has delivered the child, who was born as a girl with normal signs. The efficacy of NIPT for the detection of mosaic trisomies 21, 18 and 13 was significantly lower than that of NIPT for the detection of T21, T18 and T13. The reason for this may be due to mitotic or meiotic nondisjunction errors and trisomy rescue, thus forming mosaics, in which the multi-cellular system consisting of normal and aberrant karyotypes makes the detection of NIPT difficult, and the free fetal DNA detected by NIPT is from placental trophoblast cells, not from fetal directly. The inconsistency of mosaicism in different regions of placental tissue leads to a high number of false positives in NIPT testing [18]. Therefore, NIPT is also not recommended for screening of mosaic chromosomal abnormalities.

Among the 72 SCA samples detected, the detection rate of SCA was 0.28% and the PPV was 41.07%, among which the number of true positive cases was 3, 6, 8 and 6 for 45,X, 47,XXY, 47,XXX and 47,XYY, respectively, and their PPVs were 12.00%, 50.00%, 72.73% and 75.00%, respectively. The detection efficacy of NIPT for SCA was not as good as its efficacy for autosomal aneuploidy, with the lowest detection efficacy for 45,X (PPV: 12.00%) and moderate detection efficacy for 47,XXY, 47,XXX, and 47,XYY (PPV: 64.52%), which is in general agreement with the literature (62.70%) [19]. The high homology of X and Y chromosomes is not conducive to detection, and many segments of Y chromosome are similar to other chromosomes, which reduces the accuracy of sequencing. In addition, factors such as guanine and cytosine content bias on X chromosome, random inactivation of X chromosome, confined placental mosaicism (CPM), and maternally derived SCA can also reduce the specificity of NIPT and detection [20]. Although different equipment, Z-scores settings used in different experiments may have some influence on the test results, the efficacy of NIPT for the detection of sex chromosome triploidy is still clinically applicable, while the efficacy of 45, X is not stable. In some cases of SCA, there is often no obvious clinical phenotype in the fetus, and ultrasound does not show obvious abnormalities, so follow-up in the fetus and infancy does not reveal phenotypic abnormalities, and only after puberty does it cause serious physical and psychological effects. Therefore, prenatal screening, genetic counseling and long-term follow-up of SCA are particularly important [21], in addition, low fetal fraction is an important reason for the limitations of NIPT testing. Maternal age, weight, gestational age, tumor, and fetal placental mosaicism all influence fetal fraction, and there is wide variation among individuals. Technical factors, such as specimen collection and laboratory operation can also have an effect on it, but the threshold value is usually between 2 and 4%, and if the fetal fraction is below the lower limit of this laboratory, a "no call" signal may be given, resulting in inaccurate NIPT detection [22].

This study showed that the efficacy of NIPT for detecting other chromosomal aneuploidy was low and had obvious limitations. NIPT suggested chromosomal abnormalities in 34 cases, with 3 true-positive cases, 21 false-positive cases, and 10 cases without further verification. T7 was detected most frequently, followed by T8, T10, and T16, similar to those reported in the literature (T7, n = 6) [23], the overall PPV for other chromosomal aneuploidy was 12.50%, which was significantly lower than PPV for autosomal aneuploidy and sex chromosome aneuploidy. The high incidence of false positives may be due to the very low incidence of these rare chromosomal trisomies, as well as the possibility of uniparental disomy (UPD) due to the "trisomy rescue mechanism" during embryogenesis, CPM, maternal cell contamination, maternal tumors, and other factors [24, 25]. The three true-positive samples were T2, T9 and T16, one each. The number of sample T2 was AB198R51420, 43 years old, with 20^+5^ weeks' gestation, and karyotype of amniotic fluid cells was 46XN. The verified result of CMA was 30% mosaic trisomy of chromosome 2 with normal fetal ultrasound. The reported abnormal phenotypes were oligohydramnios, abnormal facial features, congenital heart anomalies, etc. There were also reports of some fetuses with no significant abnormal pregnancy outcome that chose to continue the pregnancy after genetic counseling, and the fetuses were born with normal phenotypes at follow-up. The number sample T16 was AB19HG04493, 33 years old, with 15^+5^ weeks's gestation, verified by CNV-Seq with seq[hg19] dup(16) (p13.3q24.3) duplication 90.35 Mb. After database search, chromosome 16 trisomy showed symptoms of intrauterine growth restriction (IUGR) as well as congenital heart defect, and after genetic counseling, the case chose to continue the pregnancy by combining with high-level ultrasound monitoring. The number of sample T9 was AB19HG06905, 28 years old, with 16^+4^ weeks' gestation, verified by karyotypes of amniotic fluid cells and CMA as: (46,XX[31]/47,XX, + 9[59]);CMA result showed 60–70% trisomy duplication of chromosome 9, and the phenotype of the result was developmental delay, cognitive impairment, microphthalmia, cardiac abnormalities, and abnormal skeletal development according to the database. Genetic counseling indicated that could cause disease. The sample opted for direct induction of labor. Therefore, results of other chromosomal aneuploidies detected using NIPT shall be integrated with routine maternal examination and fetal ultrasound screening, and information that can help in result determination, genetic counseling and clinical decision making should be provided as much as possible, which can reduce maternal anxiety and unnecessary pregnancy termination. Liang et al. [26] reported a low detection rate of other chromosomal aneuploidies by extended NIPT screening in nearly 100,000 samples with a PPV of 28.6%, which the authors suggested might also be related to the low overall incidence of the above chromosomal abnormalities and CPM. In 2016, the American College of Medical Genetics and Genomics (ACMG) stated that NIPT is not suitable for the detection of autosomal aneuploidy other than T21, T18, and T13 [27]. Therefore, the accuracy of NIPT as a test for other fetal chromosomal aneuploidies is insufficient, and the use of NIPT for the detection of other chromosomal aneuploidies should be fully informed to pregnant women about its limitations, and its results need further validation.

In the detection of CNV by using NIPT, 104 samples were detected, among which, 18 were true positive, with a PPV of 32.14%, and 38 were false positive. NIPT has certain detection efficacy for detecting CNV, but there are still many false positive cases, and the results still need further validation by karyotype analysis or CMA testing [28, 29], which is especially important for cases with pathogenic or potentially pathogenic CNV. However, despite the higher chance of false positives for CNV using NIPT, since it is difficult to detect CNV below 10 Mb by conventional karyotype analysis, NIPT can compensate for the deficiency of karyotype analysis and reduce the missed diagnosis caused by visual judgment. The results of this study showed that the NIPT had weak efficacy for CNV detection, which may be associated with the refusal from some samples to undergo further CMA validation, as well as the interference of low fetal free DNA levels, fetal placenta chimerism, and maternal-derived chromosome copy number abnormalities [30, 31]. Therefore, when the fetus is detected with CNV by NIPT, the medical history of the pregnant woman shall be further understood, and the need for interventional prenatal diagnosis shall be considered together with the results of prenatal ultrasound, and the detection rate of abnormal chromosomes shall be further improved by combining chromosome karyotyping and CMA detection techniques [32, 33]. Fetal parental chromosomes shall be selected for control analysis when necessary. Among the true positive CNV samples in this study, 5 cases were pathogenic, 4 cases were probable pathogenic, and 9 cases were of VOUS. Sample 19AH05194: NIPT reported the presence of duplication of 2.5 Mb at the 3p26.3, and the CMA result was arr[hg19]3p26.3 (285,856–2,499,708) × 3 involving 3 OMIM genes including CHL (607416), CNYN (607220) and CNTN (607280). Some patients with autism, cognitive impairment, and epilepsy were identified in the PUBMED database with a duplication of this chromosomal region, which is a possible cause of the disease. After receiving genetic counseling, the case chose to continue the pregnancy and the fetus is now born with normal growth and development, and the parents refused to perform carrier detection. Sample 20AH01147 (Fig. 1): The CMA result showed that 2q13 deletion was 2.12 Mb, which is probably pathogenic and consistent with her mother's CMA test sample (peripheral blood, No. CMA20200417), which was inherited from her mother, and the pregnancy was continued. The case chose to continue the pregnancy and the fetus has been born with normal growth and development. Sample 20AH00928: No karyotype was done, and ultrasound showed that the fetus had left hydronephrosis with dilated left ureter, permanent left superior vena cava, and widened coronary sinus. CMA analysis showed that chromosome 8 had multisegmental abnormalities, region 8p23.1p12 deletion involved 8p23.1 deletion syndrome, and typical phenotypes were congenital heart abnormalities, kidney abnormalities, developmental delay, mental retardation; the duplication at 8p12q24.3 involved partial trisomy 8q, with phenotypes of mental retardation, peculiar facial features, and cardiac anomalies, and was pathogenic CNV. After genetic counseling, the pregnant woman chose to induce labor. Sample 20AH02846 (Fig. 2): The amniotic fluid karyotype was 46,XX, del(5) (p14), and the microarray result was 5p15.33-p14.3 with 22.5 Mb deletion, involving 56 OMIM genes, and the deletion was related to Cri-du-chat syndrome. The main phenotypes included mental retardation, speech disorder, catcalling cry in infancy, etc. The deletion was pathogenic CNV. No abnormalities were found in the peripheral blood karyotype of the parents and the birth was induced after genetic counseling. Sample AB198R02189: The CMA result was 1q21.1q21.2 with 1.71 Mb duplication, involving 13 OMIM genes, and this regional duplication was associated with 1q21.1 microduplication syndrome. The main phenotypes were mild to moderate mental retardation, autism, hyperactivity, and macrosomia, etc. The syndrome was incomplete penetrance, with a penetrance of 29.1%, and the duplication was pathogenic CNV. After genetic counseling, the pregnancy was chosen to be terminated. Therefore, it is clear from the above sample analysis that NIPT is clinically relevant for the detection of CNV with clear pathogenicity and can provide an important basis for subsequent karyotyping or CMA testing [34]. In recent years, the extended NIPT test has helped to further improve the efficacy of detection of microdeletion and microduplication syndromes (MMS), but the 2015 ISPD update guidelines still limit NIPT to the definite chromosome microdeletion and microduplication syndrome [35].

## Conclusions

In conclusion, NIPT, as an important method of prenatal screening, has a high accuracy in screening for trisomies 21, 18 and 13, which have a high prevalence, especially for trisomy 21, and also has a certain reference value for the detection of SCA and CNV, which can provide a reference basis for subsequent karyotyping and chromosome microarray analysis, but is not recommended for the detection of other chromosomal aneuploidies. We believe that the detection efficacy of NIPT may be further enhanced in the future by upgrading the sequencing technology process, improving bioinformatic analysis algorithms and library construction, collecting fetal free DNA with high precision, and increasing the degree of genome-wide coverage. Only by combining maternal history examination, serological and ultrasonographic auxiliary examinations, karyotype analysis, fluorescence in situ hybridization (FISH) technology, and CMA detection technology can the advantages of NIPT technology be better utilized.

## Data Availability

The data supporting the conclusions of this article is included within the article.
